# Editorial – the state of the journal

**DOI:** 10.3402/ijch.v73.24020

**Published:** 2014-02-27

At the time of writing, the New Year has started according to the Gregorian, Julian and Lunar calendars. It is perhaps not too late to wish our readers and contributors a successful new year in 2014.

Our journal continues to prosper, thanks to the many people who publish their work in it, those who review manuscripts and those who are involved in the editorial and production processes. Our Thomson Impact Factor is inching its way up, from 1.06 in 2011 to 1.303 in 2012. The impact factor is a measure of something, arguably of quality and importance, but at least it is moving in the right direction.

The most significant change in journal policy – I hesitate to call it innovation – was the introduction of a €500 (for Europe) or $660 USD (rest of the world) fee per article accepted for publication, starting in January 2013. Exemption is possible, primarily for manuscripts from developing countries (none of which are in the Arctic), by application to our publisher Co-Action. The assessment of ability to pay is completely separate from the editorial process. The number of submissions has declined from an all-time high of 103 in 2011, to 82 in 2012 and to 56 in 2013. Whether there is a cause-and-effect relationship to the publication fee is difficult to determine, as any epidemiologist can confirm.

We have had some interesting statistics to share with our readers. The acceptance rate for articles submitted in 2013 is 57%, virtually unchanged from 2012. These figures, however, are a far cry from percentages in the high 80s in the later years of the previous decade.

Since our move from print to an exclusively online journal, beginning in early 2012, the “waiting time” has been much reduced. For articles published in 2013, the average time elapsed from receipt of a manuscript to acceptance is now down to 76 days, compared to over 200 days in the early part of this decade. The average time from receipt to publication in 2013 is 123 days, compared to 382 days in 2011.

As expected, almost all our authors are from the circumpolar countries. Based on the nationality of the corresponding author, Canada led the pack with 26%, followed by Denmark/Greenland (21%), Russia (19%), USA and Norway (9% each), Sweden (8%) and Finland (4%). There was one paper each from India and Belgium.

Enough statistics. Like books, journals must be “good reads” or we lose our readers to the numerous other diversions that are available. One is now constantly being bombarded by the appearance of new online journals. The editors will not compromise on scientific quality, but we are also looking for reports on studies that are innovative, interesting, relevant and even controversial.

And finally, we sincerely thank all the reviewers who took time out of their busy schedules to review manuscripts, without whose dedicated service there would have been no journal.

Three cheers to our reviewers:


Stig Andersen
Laura Arbour
Nick Ashbolt
Kenneth Asplund
Karen Bakker
James Berner
Peter Bjerregaard
Bert Boyer
Richard Brown
Mike Bruce
Liviana Calzavara
Sally Carraher
Susan Chatwood
Rajiev Cherukupalli
Mary Chipman
Melany Cueva
Inger Dahl-Petersen
Shelley Deeks
Anne Fanning
Leanne Findlay
Jeppe Friborg
Chris Furgal
Joanna Gaines
Tom Gleeson
Nicholas Gray
Gwen Healey
Robert Hegele
Barbara Howard
Tiina Ikäheimo
Susan Jack
Charlotte Jeppesen
Rhonda Johnson
Lawrence Joseph
Tommi Karki
James Kellner
Janet Kelly
Michael Kral
Harriet Kuhnlein
Juhani Leppäluoto
Monica Lind
Donna Manca
Robert Mann
Loraine Marrett
Paul McDonald
Florence Myrick
Sioban Nelson
Carmina Ng
Lindasay Nicolle
Nina Nielsen
Gudrun Nilsen
Peter Nilsson
Lena Maria Nilsson
Jon Odland
Pamela Orr
Larry Palinkas
David Phillips
Angela Pringle
Pernille Rønn
Charlotte Reading
Ashley Roberts
Celia Rodd
Iwona Rudkowska
Torkjel Sandanger
Amanda Sheppard
Peter Skold
Audrey Steenbeek
Vigdis Stordahl
Rosemary Waring
Sonia Wesche
Lisa Wexler

